# Supplementary material for: Target Capture Sequencing Unravels Rubus Evolution
Source: Front Plant Sci. 2019 Dec 20;10:1615. doi: 10.3389/fpls.2019.01615 (PMC6933950; doi:10.3389/fpls.2019.01615)
Supplement: Supplementary file 1 [file DataSheet_1.docx]

Supplementary Tables 1 through 8

Supplementary Table S1. Accessions of *Rubus* species and the outgroup (*Waldsteinia fragarioides*) used in this study. Species marked with an asterisk in the "Species" column did not sequence well and were not included in the results. Subgenera classifications in Focke and the USDA GRIN network are reported. Subgenera marked with an asterisk in the "USDA GRIN Subgenus Classification" column are not listed in GRIN. Current classifications were curated from other publications (Sutherland et al., 2005, Gupta and Dash, 2015, Bean, 1995, Barneby, 1988, Romoleroux, 1996). Herbarium vouchers with collector, number, and herbarium (Holmgren, Holmgren, and Barnett, 1990) or PI numbers for accessions of plants housed in the living collection at USDA NCGR Corvallis are given. MOR refers to the living collection at Morton Arboretum, Lisle, IL. HPDL refers to the Native Hawaiian Plants DNA library (Morden, Caraway, and Motley, 1996). The geographic origin for each accession is listed by continent or region. Ploidy data was collected from flow cytometry data, multiple publications, and the Missouri Botanical Garden index of plant chromosome number database (IPCN, Thompson 1995, Thompson 1997, Meng and Finn, 2002, Hummer et al., 2016). Eight major phylogenetic groups were identified in nuclear sequence analyses. The group in which each species is found is listed.

| **Species** | **Ploidy** | **USDA GRIN Subgenus Classification** | **Focke Subgenus Classification** | **Region of Origin** | **Group (1-8)** | **Voucher** |
| --- | --- | --- | --- | --- | --- | --- |
| *R. deliciosus Torr.* | 2*x* | Anoplobatus | Anoplobatus | North America | 2 | 1021, NCGR/Alice 98-1, MAINE |
| *R. odoratus* L. | 2*x* | Anoplobatus | Anoplobatus | North America | 2 | Alice R14, MAINE |
| *R. parviflorus* Nutt. | 2*x* | Anoplobatus | Anoplobatus | North America | 2 | PI 553785/CRUB 13.001 |
| *R. trilobus* Thunb. | 2*x* | Anoplobatus | Anoplobatus | South America | 2 | Ruiz 889, MO |
| *R. calycinus* Wall. Ex D. Don | 6*x* | Chamaebatus | Chamaebatus | Asia | 5 | Alice, Sutherland and Dorji from Bhutan in Alice, Sutherland and Dorji (2008) from Rubus-Ribes 2005. Now Vouchered at WKU 04-07 |
| *R. nivalis* Douglas | 2*x* | Chamaebatus | Chamaebatus | North America | 8 | 1374, NCGR/Alice 98-3, MAINE |
| *R. pectinellus* Maxim.*** | 6x | Chamaebatus | Chamaebatus | Asia | n/a | Jutila & Fujino 680, MO |
| *R. pectinarioides* | 4*x* | Chamaebatus* | n/a | Asia | 5 | Alice, Sutherland and Dorji from Bhutan in Alice, Sutherland and Dorji (2008) from Rubus-Ribes 2005. Vouchered WKU 04-25 |
| *R. sengorensis* | 4*x* | Chamaebatus* | n/a | Asia | 5 | Alice, Sutherland and Dorji from Bhutan in Alice, Sutherland and Dorji (2008) from Rubus-Ribes 2005. Vouchered WKU 04-33 |
| *R. chamaemorus* L. | 8*x* | Chamaemorus | Chamaemorus | North America/Northern Europe | 1 | Alice R17, MAINE |
| *R. geoides* Sm. | 4*x* | Comaropsis | Comaropsis | South America | 8 | Dudley et al. 1538a, MO |
| *R. arcticus* L. | 2*x* | Cylactis | Cylactis | North America/Northern Europe | 3 | T. Eriksson 701, S |
| *R. humulifolius* C. A. Mey. | 4*x* | Cylactis | Cylactis | Asia | 4 | 1173, NCGR/Alice 98-4, MAINE |
| *R. saxatilis* L. | 4*x* | Cylactis | Cylactis | Europe/Asia | 7 | 918, NCGR |
| *R. lasiococcus* A. Gray | 2*x* | Cylactis | Dalibarda | North America | 1 | Merello et al. 827, MO |
| *R. pedatus* Sm. | 2*x* | Cylactis | Dalibarda | North America/Asia | 1 | Alice 96-1, MAINE |
| *R. fockeanus* Kurz | 4*x* | Cylactis | Dalibarda | Asia | 5 | PI 606537/CRUB 1960.000 SD |
| *R. pubescens* Raf. | 2*x* | Cylactis | Eubatus | North America | 3 | Alice R15, MAINE |
| *R. treutleri* Hook. f. | 4*x* | Dalibardastrum | Dalibardastrum | Asia | 5 | Alice, Sutherland and Dorji from Bhutan in Alice, Sutherland and Dorji (2008) from Rubus-Ribes 2005. Now Vouchered at WKU 04-09 |
| *R. tricolor* Focke | 4*x* | Dalibardastrum | Dalibardastrum | Asia | 5 | Alice 97-2, MAINE |
| *R. amphidasys* Focke | 6*x* | Dalibardastrum | Malachobatus | Asia | 5 | PI 618397/CRUB 1693.001 PL |
| *R. nepalensis* (Hook.f) Kuntze | 4*x* | Dalibardastrum | n/a | Asia | 5 | Alice 97-1, MAINE |
| *R. gunnianus* Hook. | 4*x* | Diemenicus | Dalibarda | Australia | 8 | Wells 96-1, MAINE |
| *R. trifidus* Thunb. | 2*x* | Idaeobatus | Anoplobatus | Asia | 4 | 3, NCGR/ Alice 98-2, MAINE |
| *R. parvifolius* L. | 2*x* | Idaeobatus | Eubatus | Asia | 7 | PI 553813/CRUB 5.001 PL |
| *R. hawaiensis* A. Gray | 2*x* | Idaeobatus | Idaeobatus | North America (Hawaii) | 3 | 399, NCGR/Alice 98-7, MAINE |
| *R. spectabilis* Pursh | 2*x* | Idaeobatus | Idaeobatus | North America | 3 | PI 553980/CRUB 4.001 PL |
| *R. crataegifolius* Bunge | 2*x* | Idaeobatus | Idaeobatus | Asia | 4 | 16, NCGR/Alice 98-6, MAINE |
| *R. ellipticus* Sm. | 2*x* | Idaeobatus | Idaeobatus | Asia | 4 | PI 553190/CRUB 1052.001 PL |
| *R. illecebrosus* Focke | 2*x* | Idaeobatus | Idaeobatus | Asia | 4 | PI 553643/CRUB 838.001 PL |
| *R. palmatus* Thunb. | 2*x* | Idaeobatus | Idaeobatus | Asia | 4 | PI 553782/CRUB 2.002 PL |
| *R. rosifolius* Sm. | 2*x* | Idaeobatus | Idaeobatus | Asia | 4 | Eurard 11660, MO |
| *R. pentagonus* Wall. Ex Focke | 4*x* | Idaeobatus | idaeobatus | Asia | 5 | Alice, Sutherland and Dorji from Bhutan in Alice, Sutherland and Dorji (2008) from Rubus-Ribes 2005. Vouchered WKU 04-06 |
| *R. thomsonii* Focke | 4*x* | Idaeobatus | idaeobatus | Asia | 5 | Alice, Sutherland and Dorji from Bhutan in Alice, Sutherland and Dorji (2008) from Rubus-Ribes 2005. Vouchered WKU 04-31 |
| *R. alexeterius* Focke | 2*x* | Idaeobatus | Idaeobatus | Asia | 7 | Alice, Sutherland and Dorji from Bhutan in Alice, Sutherland and Dorji (2008) from Rubus-Ribes 2005. Vouchered WKU 04-23 |
| *R. coreanus Miq.* | 2*x* | Idaeobatus | Idaeobatus | Asia | 7 | PI 618447/CRUB 1438.001 PL |
| *R. idaeus* L. | 2*x* | Idaeobatus | Idaeobatus | Europe/Asia | 7 | T. Eriksson 735, S |
| *R. innominatus* S. Moore | 2*x* | Idaeobatus | Idaeobatus | Asia | 7 | PI 553646/CRUB 1039.001 PL |
| *R. lasiostylus* Focke | 2*x* | Idaeobatus | Idaeobatus | Asia | 7 | PI 553668/CRUB 425.001 PL |
| *R. leucodermis* Douglas ex Torr. & A. Gray | 2*x* | Idaeobatus | Idaeobatus | North America | 7 | PI 553673/CRUB 14.001 PL |
| *R. niveus* Thunb. | 2*x* | Idaeobatus | Idaeobatus | Asia | 7 | PI 553723 |
| *R. occidentalis* L. | 2*x* | Idaeobatus | Idaeobatus | North America | 7 | AliceR16,MAINE |
| *R. phoenicolasius* Maxim. | 2*x* | Idaeobatus | Idaeobatus | Asia | 7 | Alice96-2,MAINE |
| *R. pungens* Cambess. | 2*x* | Idaeobatus | Idaeobatus | Asia | 7 | PI 553849/CRUB 46.002 PL |
| *R. sachalinensis* H. Lév. | 4*x* | Idaeobatus | Idaeobatus | Asia | 7 | PI 553866/CRUB 626.001 PL |
| *R. strigosus* Michx. | 2*x* | Idaeobatus | Idaeobatus | North America | 7 | Maine Alice R8 |
| *R. macraei* A. Gray | 6*x* | Idaeobatus | n/a | North America (Hawaii) | 6 | Gardners. n. , HPDL207 |
| Logan | 6*x* | Idaeorubus | n/a | Cultivar | 7 | PI553258/CRUB 81.001 PL |
| Boysen | 7*x* | Idaeorubus | n/a | Cultivar | 8 | PI553341/CRUB 1108.001 |
| Marion | 6*x* | Idaeorubus | n/a | Cultivar | 8 | PI553254/CRUB 385.001 |
| *R. assamensis* Focke | 4*x* | Malachobatus | Malachobatus | Asia | 5 | 1701, NCGR |
| *R. ichangensis* Hemsl. & Kuntze | 4*x* | Malachobatus | Malachobatus | Asia | 5 | PI 618453/CRUB 1606.001 PL |
| *R. irenaeus* Focke | 6*x* | Malachobatus | Malachobatus | Asia | 5 | PI 618550/CRUB 1607.001 PL |
| *R. lambertianus* Ser. | 4*x* | Malachobatus | Malachobatus | Asia | 5 | Boufford & Bartholomew 23955, MO |
| *R. lineatus* Reinw. | 4*x* | Malachobatus | Malachobatus | Asia | 5 | Grierson & Long 1950, GH |
| *R. clinocephalus* Focke | 4*x* | Malachobatus | Malachobatus | Asia | 5 | PI 606459 |
| *R. tephrodes* Hance | 4*x* | Malachobatus | Malachobatus | Asia | 5 | Yao 9231, MO |
| *R. australis* G. Forst. | 4*x* | Micranthobatus | Lampobatus | New Zealand | 8 | Gardner 1539, MO |
| *R. parvus* Buchanan | 4*x* | Micranthobatus | Lampobatus | New Zealand | 8 | Alice 97-3, MAINE |
| *R. moorei* F. Muell. | 4*x* | Micranthobatus* | Lampobatus | Australia | 8 | Streimann 8207, GH |
| *R. calophyllus* | 4*x* | n/a | Malachobatus | Asia | 5 | Alice, Sutherland and Dorji from Bhutan in Alice, Sutherland and Dorji (2008) from Rubus-Ribes 2005. Vouchered WKU 04-24 |
| *R. repens* (L.) Kuntze | 2*x* | n/a | Dalibarda | North America | 1 | Alice 97-4, MAINE |
| *R. ursinus x*  *R. armeniacus(1)* | 8*x* | n/a | n/a | North America | 8 | Alice personal collection |
| *R. ursinus x*  *R. armeniacus(6)* | 8*x* | n/a | n/a | North America | 8 | Alice personal collection |
| *R. acanthophyllus* Focke | 6*x* | n/a | Orobatus | South America | 8 | Alice and Cantrell are collectors in Ecuador WKU 07-11 |
| *W. fragarioides* (Michx.) Tratt. | 2*x* | n/a | n/a | North America | Outgroup | Hill & Soblo 21384, GH |
| *R. glabratus* Kunth | 6*x* | Orobatus | Orobatus | South America | 8 | PI 548901/CRUB 1251.004 PL |
| *R. loxensis Benth.* | 6*x* | Orobatus | Orobatus | South America | 8 | Alice and Cantrell are collectors in Ecuador WKU 07-17 |
| *R. roseus* Poir. | 6*x* | Orobatus | Orobatus | South America | 8 | Luteyn & Quezada 14402, MO |
| *R. laegaardii* Romol. | 6*x* | Orobatus* | n/a | South America | 8 | Alice and Cantrell are collectors in Ecuador Voucher WKU 07-15 |
| *R. hispidus* L. *** | 2*x* | Rubus | Eubatus | North America | n/a | Alice R9, MAINE |
| *R. caesius* L. | 4*x* | Rubus | Eubatus | Europe/Asia | 6 | Karlen 243, S |
| *R. ursinus* Cham. Et. Schltdl. *(2)* | 8*x* | Rubus | Eubatus | North America | 6 | PI 604641 |
| *R. ursinus(3)* | 12*x* | Rubus | Eubatus | North America | 6 | PI 554067 |
| *R. ursinus(4)* | 13*x* | Rubus | Eubatus | North America | 6 | USDA Accession no longer exists |
| *R. ursinus(5)* | 6*x* | Rubus | Eubatus | North America | 6 | PI 604641 |
| *R. allegheniensis* Porter | 2*x* | Rubus | Eubatus | North America | 8 | Alice R1, MAINE |
| *R. argutus* Link | 2*x* | Rubus | Eubatus | North America | 8 | Alice & Judd 15, MAINE |
| *R. armeniacus* Focke | 4*x* | Rubus | Eubatus | Europe/Asia | 8 | PI 618579/CRUB 45.001 PL |
| *R. bifrons* Vest | 4*x* | Rubus | Eubatus | Europe/Asia | 8 | Alice 98-9, MAINE |
| *R. canadensis* L. | 2*x* | Rubus | Eubatus | North America | 8 | Alice & Campbell 98-10, MAINE |
| *R. caucasicus* Focke | 4*x* | Rubus | Eubatus | Europe/Asia | 8 | PI 553143/CRUB 54.001 PL |
| *R. coriifolius* Liebm. | 2*x* | Rubus | Eubatus | Americas | 8 | Alice and Dodson from Mexico. Vouchered WKU 06-05 |
| *R. cuneifolius* Pursh | 2*x* | Rubus | Eubatus | North America | 8 | Alice 5, MAINE |
| *R. flagellaris* Willd. | 4-9*x* | Rubus | Eubatus | North America | 8 | PI 553787 |
| *R. laciniatus* Willd. | 4*x* | Rubus | Eubatus | Europe/Asia | 8 | PI 618548/CRUB 1596.001 PL |
| *R. robustus* C. Presl | 2*x* | Rubus | Eubatus | Americas | 8 | Steinbach 247, GH |
| *R. setosus* Bigelow | 2*x* | Rubus | Eubatus | North America | 8 | Alice 113, MAINE |
| *R. trivialis* Michx. | 2*x* | Rubus | Eubatus | North America | 8 | Alice 33, MAINE |
| *R. ulmifolius* Schott | 2*x* | Rubus | Eubatus | Europe/Asia | 8 | 190-84, MOR |
| *R. urticifolius* Poir. | 2*x* | Rubus | Eubatus | Americas | 8 | PI 548929/CRUB 1288.001 PL |
| *R. glaucus* Benth. | 4*x* | Rubus | Idaeobatus | South America | 6 | PI 548906/CRUB 1293.001 PL |
| *R. eriocarpus* Liebm. | 2*x* | Rubus | Idaeobatus | South America | 7 | Alice and Dodson from Mexico. Vouchered WKU 06-12 |
| *R. pensilvanicus* Poir. | 4*x* | Rubus | n/a | North America | 8 | Alice R5, MAINE |

Supplementary Table S2. Success of target capture for each *Rubus* sample. Statistics except average read depth calculated with HybPiper script hybpiper_stats.py. Total number of target genes is 1173. Samples marked with an asterisk did not sequence well and were not included in phylogenetic analyses.

| **­** | **Number of reads (Mbp)** | **Reads mapped to targets (Millions)** | **% Reads on target** | **Number of target genes with sequences** | **Number of target sequences > 75% of target length** | **Average read depth over all targets** |
| --- | --- | --- | --- | --- | --- | --- |
| **Average** | **2.7** | **1.9** | **72%** | **1090** | **988** | **66.8** |
| Boysen | 3.0 | 2.0 | 67% | 1140 | 1054 | 71.1 |
| Logan | 5.3 | 3.9 | 74% | 1149 | 1119 | 137.9 |
| Marion | 2.6 | 1.9 | 74% | 1145 | 1087 | 66.2 |
| *R. acanthophyllus* | 4.0 | 3.3 | 81% | 1148 | 1103 | 114.2 |
| *R. alexeterius* | 1.4 | 1.2 | 87% | 1140 | 1065 | 42.6 |
| *R. allegheniensis* | 0.2 | 0.2 | 72% | 778 | 66 | 6.0 |
| *R. amphidasys* | 4.9 | 3.1 | 63% | 1146 | 1094 | 110.0 |
| *R. arcticus* | 3.0 | 2.4 | 80% | 1146 | 1087 | 83.3 |
| *R. argutus* | 3.3 | 2.4 | 73% | 1141 | 1083 | 85.6 |
| *R. armeniacus* | 1.3 | 1.0 | 74% | 1110 | 929 | 33.8 |
| *R. assamensis* | 7.1 | 5.5 | 77% | 1143 | 1104 | 192.0 |
| *R. australis* | 7.0 | 5.5 | 79% | 1148 | 1097 | 194.6 |
| *R. bifrons* | 2.0 | 1.5 | 75% | 1133 | 1039 | 53.5 |
| *R. caesius* | 5.6 | 3.5 | 62% | 1148 | 1102 | 122.2 |
| *R. calophyllus* | 2.1 | 1.6 | 79% | 1137 | 1033 | 57.7 |
| *R. calycinus* | 1.5 | 1.2 | 78% | 1134 | 994 | 41.6 |
| *R. canadensis* | 4.9 | 3.6 | 73% | 1146 | 1092 | 125.8 |
| *R. caucasicus* | 1.1 | 0.9 | 84% | 1126 | 999 | 32.5 |
| *R. chamaemorus* | 0.6 | 0.5 | 84% | 1081 | 639 | 17.3 |
| *R. coreanus* | 4.7 | 3.8 | 80% | 1152 | 1112 | 132.2 |
| *R. coriifolius* | 1.2 | 0.9 | 70% | 1101 | 902 | 30.5 |
| *R. crataegifolius* | 4.2 | 2.7 | 64% | 1143 | 1077 | 93.9 |
| *R. cuneifolius* | 2.3 | 1.8 | 77% | 1138 | 1055 | 61.5 |
| *R. deliciosus* | 2.0 | 1.5 | 74% | 1139 | 1064 | 52.1 |
| *R. ellipticus* | 2.2 | 1.7 | 75% | 1141 | 1070 | 58.1 |
| *R. eriocarpus* | 3.1 | 2.5 | 80% | 1148 | 1098 | 88.2 |
| *R. flagellaris* | 5.9 | 4.8 | 81% | 1149 | 1121 | 167.1 |
| *R. fockeanus* | 5.4 | 3.8 | 70% | 1149 | 1108 | 132.0 |
| *R. geoides* | 5.4 | 4.1 | 77% | 1152 | 1111 | 144.5 |
| *R. glabratus* | 5.1 | 3.4 | 67% | 1147 | 1106 | 121.1 |
| *R. glaucus* | 1.4 | 1.1 | 77% | 1119 | 986 | 36.9 |
| *R. gunnianus* | 2.9 | 2.1 | 74% | 1140 | 1074 | 75.4 |
| *R. hawaiensis* | 1.3 | 1.0 | 81% | 1127 | 1009 | 35.6 |
| *R. hispidus** | 0.0 | 0.0 | 82% | 3 | - | 0.4 |
| *R. humulifolius* | 1.0 | 0.8 | 83% | 1119 | 978 | 28.2 |
| *R. ichangensis* | 6.1 | 4.9 | 80% | 1146 | 1109 | 170.4 |
| *R. idaeus* | 2.9 | 2.0 | 69% | 1151 | 1099 | 71.5 |
| *R. illecebrosus* | 4.9 | 3.3 | 67% | 1143 | 1091 | 114.8 |
| *R. innominatus* | 2.0 | 1.6 | 79% | 1135 | 1063 | 54.5 |
| *R. irenaeus* | 5.2 | 3.9 | 76% | 1149 | 1103 | 138.4 |
| *R. laciniatus* | 1.9 | 1.4 | 76% | 1132 | 1038 | 50.7 |
| *R. laegardii* | 1.2 | 1.1 | 87% | 1131 | 994 | 37.0 |
| *R. lambertianus* | 2.5 | 2.0 | 79% | 1137 | 1066 | 69.2 |
| *R. lasiococcus* | 0.8 | 0.6 | 78% | 1107 | 902 | 22.2 |
| *R. lasiostylus* | 4.9 | 3.3 | 69% | 1148 | 1101 | 117.5 |
| *R. leucodermis* | 3.7 | 2.4 | 65% | 1154 | 1111 | 84.3 |
| *R. lineatus* | 2.3 | 1.8 | 80% | 1140 | 1054 | 64.3 |
| *R. loxensis* | 1.4 | 1.0 | 76% | 1112 | 946 | 36.5 |
| *R. macraei* | 3.1 | 2.5 | 81% | 1146 | 1095 | 87.3 |
| *R. moorei* | 2.5 | 2.0 | 78% | 1145 | 1057 | 69.4 |
| *R. clinocephalus* | 1.4 | 1.1 | 79% | 1120 | 979 | 38.4 |
| *R. nepalensis* | 0.3 | 0.2 | 81% | 620 | 89 | 7.6 |
| *R. nivalis* | 0.8 | 0.7 | 80% | 1097 | 910 | 23.3 |
| *R. niveus* | 1.0 | 0.6 | 58% | 1090 | 909 | 21.1 |
| *R. occidentalis* | 2.1 | 1.2 | 59% | 1135 | 1075 | 43.6 |
| *R. odoratus* | 2.2 | 1.2 | 54% | 1135 | 1034 | 41.3 |
| *R. palmatus* | 0.3 | 0.3 | 77% | 701 | 260 | 9.1 |
| *R. parviflorus* | 1.3 | 0.9 | 73% | 1130 | 1011 | 33.2 |
| *R. parvifolius* | 1.7 | 1.2 | 69% | 1129 | 1053 | 41.7 |
| *R. parvus* | 3.0 | 1.4 | 45% | 1137 | 1037 | 48.5 |
| *R. pectinarioides* | 2.5 | 2.1 | 81% | 1142 | 1086 | 72.0 |
| *R. pectinellus** | 0.0 | 0.0 | 75% | 0 | - | 0.1 |
| *R. pedatus* | 3.8 | 3.0 | 78% | 1143 | 1104 | 105.3 |
| *R. pensilvanicus* | 1.5 | 1.2 | 82% | 1127 | 1053 | 41.7 |
| *R. pentagonus* | 1.8 | 1.3 | 76% | 1130 | 984 | 46.9 |
| *R. phoenicolasius* | 2.9 | 2.3 | 82% | 1155 | 1110 | 82.4 |
| *R. pubescens* | 1.1 | 0.7 | 67% | 1110 | 956 | 25.6 |
| *R. pungens* | 0.8 | 0.5 | 66% | 1099 | 874 | 18.5 |
| *R. repens* | 1.0 | 0.7 | 74% | 1111 | 938 | 26.3 |
| *R. robustus* | 2.6 | 2.1 | 82% | 1144 | 1091 | 74.8 |
| *R. roseus* | 3.3 | 2.4 | 72% | 1146 | 1090 | 84.1 |
| *R. rosifolius* | 4.5 | 3.3 | 75% | 1145 | 1081 | 117.0 |
| *R. sachalinensis* | 4.3 | 3.2 | 75% | 1151 | 1114 | 111.8 |
| *R. saxatilis* | 4.2 | 2.6 | 62% | 1152 | 1105 | 91.9 |
| *R. sengorensis* | 5.3 | 2.2 | 41% | 1142 | 1073 | 75.5 |
| *R. setosus* | 4.7 | 2.3 | 48% | 1137 | 1090 | 79.1 |
| *R. spectabilis* | 3.6 | 2.0 | 54% | 1145 | 1098 | 69.5 |
| *R. strigosus* | 7.7 | 3.4 | 45% | 1155 | 1127 | 120.7 |
| *R. tephrodes* | 1.1 | 0.8 | 75% | 1111 | 913 | 28.3 |
| *R. thomsonii* | 0.9 | 0.7 | 70% | 1082 | 789 | 22.9 |
| *R. treutleri* | 0.9 | 0.7 | 72% | 1092 | 848 | 23.9 |
| *R. tricolor* | 0.7 | 0.4 | 58% | 1023 | 581 | 14.9 |
| *R. trifidus* | 1.5 | 0.6 | 44% | 1098 | 929 | 22.2 |
| *R. trilobus* | 2.6 | 1.7 | 68% | 1143 | 1080 | 61.5 |
| *R. trivialis* | 0.6 | 0.3 | 58% | 992 | 526 | 12.0 |
| *R. ulmifolius* | 3.0 | 2.0 | 66% | 1144 | 1095 | 69.7 |
| *R. ursinus* x *R. armeniacus 1* | 4.2 | 3.2 | 75% | 1149 | 1116 | 111.0 |
| *R. ursinus2* | 1.0 | 0.8 | 78% | 1122 | 908 | 27.1 |
| *R. ursinus3* | 5.8 | 3.5 | 61% | 1157 | 1125 | 123.8 |
| *R. ursinus4* | 2.0 | 1.7 | 85% | 1144 | 1070 | 59.4 |
| *R. ursinus5* | 2.0 | 1.5 | 76% | 1142 | 1055 | 54.1 |
| *R. ursinus* x *R. armeniacus 6* | 1.2 | 0.8 | 63% | 1100 | 871 | 27.3 |
| *R. urticifolius* | 1.4 | 0.7 | 49% | 1108 | 948 | 24.4 |
| *W. fragarioides* | 1.1 | 0.3 | 28% | 856 | 266 | 11.4 |

Supplementary Table S3. Alignment statistics for analyses for exon and supercontigs (S, exon + noncoding sequences) for diploid and all taxa *Rubus* datasets. Samples marked with an asterisk did not sequence well and were not included in phylogenetic analyses. E = exon sequence alignments. Ungapped lengths are reported.

| **Sample** | **% Cov. strawberry/apple/peach targets** | **% Cov. *R. occ.* targets** | **Length of S seq. alignment in All Taxa (Mbp)** | **% Non-gap bases in S seq. alignment for all taxa** | **Length of S seq. alignment in diploid taxa (Mbp)** | **% Non-gap bases in S seq. alignment for diploid taxa** | **Length of E seq. alignment in all taxa (Mbp)** | **% Non-gap bases in E seq. alignment for all taxa** | **Length of E seq. alignment in diploid taxa (Mbp)** | **% Non-gap bases in E seq. alignment for diploid taxa** |
| --- | --- | --- | --- | --- | --- | --- | --- | --- | --- | --- |
| **Average** | **86%** | **101%** | **2.7** | **27%** | **3.9** | **36%** | **1.5** | **58%** | **1.6** | **61%** |
| Boysen | 91% | 108% | 1.46 | 15% | - | - | 0.81 | 32% | - | - |
| Logan | 97% | 113% | 1.61 | 16% | - | - | 1.11 | 44% | - | - |
| Marion | 92% | 109% | 1.83 | 18% | - | - | 1.12 | 44% | - | - |
| *R. acanthophyllus* | 95% | 112% | 1.29 | 13% | - | - | 1.26 | 50% | - | - |
| *R. alexeterius* | 88% | 105% | 2.78 | 28% | 2.89 | 29% | 1.54 | 61% | 1.45 | 57% |
| *R. allegheniensis* | 18% | 27% | 0.01 | 0% | 0.03 | 0% | 0.05 | 2% | 0.04 | 1% |
| *R. amphidasys* | 96% | 111% | 1.18 | 12% | - | - | 1.38 | 54% | - | - |
| *R. arcticus* | 93% | 107% | 4.58 | 45% | 4.64 | 46% | 2.05 | 81% | 1.86 | 73% |
| *R. argutus* | 93% | 106% | 4.14 | 41% | 4.26 | 42% | 2.03 | 80% | 1.89 | 75% |
| *R. armeniacus* | 79% | 97% | 3.87 | 38% | - | - | 1.72 | 68% | - | - |
| *R. assamensis* | 98% | 112% | 0.86 | 8% | - | - | 1.20 | 47% | - | - |
| *R. australis* | 98% | 112% | 1.10 | 11% | - | - | 1.29 | 51% | - | - |
| *R. bifrons* | 90% | 103% | 3.81 | 38% | - | - | 1.88 | 74% | - | - |
| *R. caesius* | 97% | 112% | 0.93 | 9% | - | - | 0.68 | 27% | - | - |
| *R. calophyllus* | 90% | 106% | 1.17 | 12% | - | - | 1.01 | 40% | - | - |
| *R. calycinus* | 86% | 104% | 1.04 | 10% | - | - | 0.86 | 34% | - | - |
| *R. canadensis* | 95% | 107% | 3.93 | 39% | 4.11 | 41% | 1.93 | 76% | 1.83 | 72% |
| *R. caucasicus* | 85% | 99% | 3.75 | 37% | - | - | 1.74 | 69% | - | - |
| *R. chamaemorus* | 66% | 75% | 0.80 | 8% | - | - | 0.88 | 35% | - | - |
| *R. coreanus* | 95% | 110% | 4.78 | 47% | 4.85 | 48% | 1.95 | 77% | 1.76 | 69% |
| *R. coriifolius* | 78% | 94% | 3.50 | 35% | 3.57 | 35% | 1.88 | 74% | 1.64 | 64% |
| *R. crataegifolius* | 94% | 107% | 3.49 | 35% | 3.53 | 35% | 1.92 | 76% | 1.74 | 69% |
| *R. cuneifolius* | 92% | 104% | 4.53 | 45% | 4.60 | 45% | 2.09 | 83% | 1.93 | 76% |
| *R. deliciosus* | 93% | 105% | 5.13 | 51% | 5.18 | 51% | 2.11 | 83% | 1.88 | 74% |
| *R. ellipticus* | 93% | 105% | 3.16 | 31% | 3.52 | 35% | 1.43 | 57% | 1.41 | 56% |
| *R. eriocarpus* | 93% | 108% | 5.59 | 55% | 5.65 | 56% | 2.13 | 84% | 2.01 | 79% |
| *R. flagellaris* | 97% | 114% | 1.17 | 12% | - | - | 1.11 | 44% | - | - |
| *R. fockeanus* | 97% | 112% | 1.46 | 14% | - | - | 1.00 | 39% | - | - |
| *R. geoides* | 97% | 113% | 0.94 | 9% | - | - | 1.18 | 46% | - | - |
| *R. glabratus* | 96% | 113% | 1.32 | 13% | - | - | 1.34 | 53% | - | - |
| *R. glaucus* | 81% | 105% | 1.91 | 19% | - | - | 1.09 | 43% | - | - |
| *R. gunnianus* | 94% | 109% | 1.66 | 16% | - | - | 1.45 | 57% | - | - |
| *R. hawaiensis* | 86% | 102% | 4.99 | 49% | 5.06 | 50% | 2.15 | 85% | 1.90 | 75% |
| *R. hispidus** | - | - | - | - | - | - | - | - | - | - |
| *R. humulifolius* | 86% | 98% | 2.32 | 23% | - | - | 1.24 | 49% | - | - |
| *R. ichangensis* | 98% | 114% | 0.97 | 10% | - | - | 1.17 | 46% | - | - |
| *R. idaeus* | 93% | 109% | 4.61 | 46% | 4.73 | 47% | 1.93 | 76% | 1.79 | 70% |
| *R. illecebrosus* | 95% | 106% | 4.47 | 44% | 4.50 | 44% | 2.08 | 82% | 1.87 | 74% |
| *R. innominatus* | 91% | 105% | 5.11 | 51% | 5.22 | 52% | 1.93 | 76% | 1.72 | 68% |
| *R. irenaeus* | 97% | 113% | 1.37 | 14% | - | - | 1.55 | 61% | - | - |
| *R. laciniatus* | 89% | 103% | 3.64 | 36% | - | - | 1.73 | 68% | - | - |
| *R. laegardii* | 86% | 101% | 1.79 | 18% | - | - | 1.26 | 50% | - | - |
| *R. lambertianus* | 93% | 110% | 1.17 | 12% | - | - | 1.19 | 47% | - | - |
| *R. lasiococcus* | 78% | 94% | 3.23 | 32% | 3.28 | 32% | 1.76 | 70% | 1.53 | 60% |
| *R. lasiostylus* | 95% | 108% | 5.40 | 54% | 5.41 | 53% | 1.87 | 74% | 1.69 | 67% |
| *R. leucodermis* | 93% | 110% | 5.87 | 58% | 5.92 | 58% | 2.11 | 83% | 1.99 | 78% |
| *R. lineatus* | 90% | 108% | 1.36 | 14% | - | - | 1.01 | 40% | - | - |
| *R. loxensis* | 83% | 98% | 2.24 | 22% | - | - | 1.32 | 52% | - | - |
| *R. macraei* | 92% | 113% | 0.96 | 10% | - | - | 0.94 | 37% | - | - |
| *R. moorei* | 91% | 108% | 0.79 | 8% | - | - | 1.28 | 51% | - | - |
| *R. clinocephaluss* | 82% | 103% | 1.90 | 19% | - | - | 1.33 | 52% | - | - |
| *R. nepalensis* | 20% | 22% | 0.11 | 1% | - | - | 0.12 | 5% | - | - |
| *R. nivalis* | 79% | 95% | 2.89 | 29% | 3.12 | 31% | 1.38 | 54% | 1.60 | 63% |
| *R. niveus* | 75% | 96% | 3.42 | 34% | 3.44 | 34% | 1.84 | 72% | 1.57 | 62% |
| *R. occidentalis* | 87% | 107% | 5.67 | 56% | 5.75 | 57% | 2.14 | 85% | 1.97 | 77% |
| *R. odoratus* | 90% | 103% | 4.84 | 48% | 4.86 | 48% | 2.11 | 83% | 1.85 | 73% |
| *R. palmatus* | 29% | 42% | 0.77 | 8% | 0.78 | 8% | 0.56 | 22% | 0.45 | 18% |
| *R. parviflorus* | 87% | 103% | 4.21 | 42% | 4.24 | 42% | 2.02 | 80% | 1.80 | 71% |
| *R. parvifolius* | 87% | 104% | 4.49 | 44% | 4.59 | 45% | 2.02 | 79% | 1.82 | 72% |
| *R. parvus* | 90% | 106% | 1.48 | 15% | - | - | 1.33 | 52% | - | - |
| *R. pectinarioides* | 94% | 110% | 1.70 | 17% | - | - | 1.25 | 49% | - | - |
| *R. pectinellus** | - | - | - | - | - | - | - | - | - | - |
| *R. pedatus* | 95% | 108% | 4.20 | 42% | 4.25 | 42% | 1.85 | 73% | 1.68 | 66% |
| *R. pensilvanicus* | 89% | 104% | 3.54 | 35% | - | - | 1.93 | 76% | - | - |
| *R. pentagonus* | 86% | 101% | 2.14 | 21% | - | - | 1.01 | 40% | - | - |
| *R. phoenicolasius* | 93% | 110% | 4.44 | 44% | 4.52 | 45% | 1.96 | 77% | 1.73 | 68% |
| *R. pubescens* | 80% | 98% | 4.07 | 40% | 4.15 | 41% | 1.95 | 77% | 1.74 | 68% |
| *R. pungens* | 76% | 93% | 2.03 | 20% | 2.30 | 23% | 1.26 | 50% | 1.29 | 51% |
| *R. repens* | 84% | 95% | 2.23 | 22% | 2.27 | 22% | 1.42 | 56% | 1.33 | 52% |
| *R. robustus* | 94% | 106% | 4.29 | 43% | 4.37 | 43% | 2.02 | 80% | 1.87 | 74% |
| *R. roseus* | 94% | 111% | 1.39 | 14% | - | - | 1.32 | 52% | - | - |
| *R. rosifolius* | 94% | 105% | 4.53 | 45% | 4.57 | 45% | 2.07 | 81% | 1.87 | 73% |
| *R. sachalinensis* | 94% | 109% | 5.04 | 50% | - | - | 1.98 | 78% | - | - |
| *R. saxatilis* | 95% | 111% | 1.82 | 18% | - | - | 1.01 | 40% | - | - |
| *R. sengorensis* | 93% | 110% | 1.17 | 12% | - | - | 1.13 | 44% | - | - |
| *R. setosus* | 93% | 107% | 4.09 | 41% | 4.36 | 43% | 1.73 | 68% | 1.76 | 69% |
| *R. spectabilis* | 93% | 109% | 5.24 | 52% | 5.31 | 52% | 2.12 | 83% | 1.94 | 76% |
| *R. strigosus* | 96% | 111% | 5.04 | 50% | 5.14 | 51% | 2.00 | 79% | 1.81 | 71% |
| *R. tephrodes* | 78% | 96% | 1.39 | 14% | - | - | 1.25 | 49% | - | - |
| *R. thomsonii* | 71% | 86% | 2.07 | 20% | - | - | 1.02 | 40% | - | - |
| *R. treutleri* | 75% | 90% | 1.78 | 18% | - | - | 1.28 | 51% | - | - |
| *R. tricolor* | 60% | 70% | 1.17 | 12% | - | - | 0.79 | 31% | - | - |
| *R. trifidus* | 82% | 94% | 2.95 | 29% | 3.05 | 30% | 1.79 | 71% | 1.57 | 62% |
| *R. trilobus* | 93% | 107% | 5.37 | 53% | 5.40 | 53% | 2.17 | 86% | 1.97 | 78% |
| *R. trivialis* | 58% | 67% | 0.43 | 4% | 0.51 | 5% | 0.52 | 20% | 0.51 | 20% |
| *R. ulmifolius* | 93% | 106% | 3.76 | 37% | 3.86 | 38% | 2.00 | 79% | 1.80 | 71% |
| *R. ursinus* x *R. armeniacus 1* | 95% | 113% | 1.33 | 13% | - | - | 1.15 | 45% | - | - |
| *R. ursinus2* | 77% | 95% | 1.56 | 15% | - | - | 0.98 | 39% | - | - |
| *R. ursinus3* | 96% | 114% | 1.63 | 16% | - | - | 1.29 | 51% | - | - |
| *R. ursinus4* | 89% | 110% | 1.94 | 19% | - | - | 1.36 | 53% | - | - |
| *R. ursinus5* | 88% | 108% | 1.97 | 20% | - | - | 1.37 | 54% | - | - |
| *R. ursinus* x *R. armeniacus 6* | 75% | 92% | 2.81 | 28% | - | - | 1.31 | 52% | - | - |
| *R. urticifolius* | 81% | 98% | 3.54 | 35% | 3.59 | 35% | 2.03 | 80% | 1.79 | 70% |
| *W. fragarioides* | 68% | 36% | 0.02 | 0% | 0.02 | 0% | 0.07 | 3% | 0.08 | 3% |

Supplementary Table S4. Chloroplast sequence statistics. *Rubus* samples marked with an asterisk either failed sequencing or were removed from the analysis as rogue taxa by RogueNaRok. Average read depth was calculated from mapping up to 100,000 reads against the *R. occidentalis* reference chloroplast sequence edited to include one copy of the inverted repeat region.

| **Sample** | **Avg. read depth of chloroplast sequence** | **Ungapped length of the chloroplast genome (bp)** | **% Gaps in chloroplast genome** |
| --- | --- | --- | --- |
| **Average** | **23** | **124,184** | **1%** |
| Boysen | 12.6 | 124,309 | 1% |
| Logan | 37.7 | 125,567 | 0% |
| Marion | 21.4 | 125,663 | 0% |
| *R. acanthophyllus* | 11.1 | 122,694 | 2% |
| *R. alexeterius* | 7.9 | 123,719 | 2% |
| *R. allegheniensis* | 3.1 | 115,577 | 8% |
| *R. amphidasys* | 29.2 | 125,522 | 0% |
| *R. arcticus* | 17.5 | 123,400 | 2% |
| *R. argutus* | 16 | 125,537 | 0% |
| *R. armeniacus* | 5.6 | 125,441 | 0% |
| *R. assamensis* | 20.2 | 125,711 | 0% |
| *R. australis* | 6.9 | 125,838 | 0% |
| *R. bifrons* | 7.7 | 124,415 | 1% |
| *R. caesius* | 61.9 | 125,659 | 0% |
| *R. calophyllus* | 1.4 | 111,561 | 11% |
| *R. calycinus* | 6 | 121,047 | 4% |
| *R. canadensis* | 11.8 | 125,432 | 0% |
| *R. caucasicus** | 1.9 | - | - |
| *R. chamaemorus* | 1.9 | 125,640 | 0% |
| *R. coreanus* | 11.2 | 125,007 | 1% |
| *R. coriifolius* | 4.3 | 124,525 | 1% |
| *R. crataegifolius* | 59.1 | 125,125 | 1% |
| *R. cuneifolius* | 4.5 | 125,520 | 0% |
| *R. deliciosus* | 24.9 | 125,102 | 1% |
| *R. ellipticus* | 12.3 | 123,604 | 2% |
| *R. eriocarpus* | 9.6 | 124,179 | 1% |
| *R. flagellaris* | 12.6 | 122,233 | 3% |
| *R. fockeanus* | 48.8 | 125,693 | 0% |
| *R. geoides* | 16.6 | 125,418 | 0% |
| *R. glabratus* | 74.4 | 125,383 | 0% |
| *R. glaucus* | 6.5 | 125,225 | 0% |
| *R. gunnianus* | 32.2 | 125,184 | 0% |
| *R. hawaiensis* | 5.1 | 125,115 | 1% |
| *R. hispidus** | - | - | - |
| *R. humulifolius* | 9.2 | 123,176 | 2% |
| *R. ichangensis* | 27.1 | 125,688 | 0% |
| *R. idaeus* | 58 | 124,689 | 1% |
| *R. illecebrosus* | 68.2 | 124,484 | 1% |
| *R. innominatus* | 4 | 124,262 | 1% |
| *R. irenaeus* | 12 | 125,706 | 0% |
| *R. laciniatus* | 8.9 | 125,496 | 0% |
| *R. laegardii* | 3.5 | 118,943 | 5% |
| *R. lambertianus** | 6.5 | - | - |
| *R. lasiococcus* | 4.5 | 124,906 | 1% |
| *R. lasiostylus* | 47.9 | 124,685 | 1% |
| *R. leucodermis* | 76 | 125,721 | 0% |
| *R. lineatus* | 3 | 123,760 | 2% |
| *R. loxensis* | 2.8 | 125,068 | 1% |
| *R. macraei* | 8.7 | 121,732 | 3% |
| *R. moorei* | 5 | 124,514 | 1% |
| *R. clinocephaus* | 2.6 | 125,129 | 1% |
| *R. nepalensis* | 3.4 | 124,770 | 1% |
| *R. nivalis* | 7.1 | 121,338 | 4% |
| *R. niveus* | 14.4 | 125,501 | 0% |
| *R. occidentalis* | 24.9 | 125,769 | 0% |
| *R. odoratus* | 41.8 | 125,620 | 0% |
| *R. palmatus* | 1.6 | 125,003 | 1% |
| *R. parviflorus* | 19 | 125,587 | 0% |
| *R. parvifolius* | 14.8 | 125,386 | 0% |
| *R. parvus* | 75.5 | 125,463 | 0% |
| *R. pectinarioides* | 7.1 | 125,435 | 0% |
| *R. pectinellus** | - | - | - |
| *R. pedatus* | 49 | 125,696 | 0% |
| *R. pensilvanicus* | 7.1 | 124,309 | 1% |
| *R. pentagonus* | 3.8 | 125,580 | 0% |
| *R. phoenicolasius* | 26.3 | 125,140 | 1% |
| *R. pubescens* | 8.5 | 125,487 | 0% |
| *R. pungens* | 16.5 | 125,668 | 0% |
| *R. repens* | 3.1 | 111,902 | 11% |
| *R. robustus** | 8.3 | - | - |
| *R. roseus* | 17.1 | 125,430 | 0% |
| *R. rosifolius* | 17.8 | 124,949 | 1% |
| *R. sachalinensis* | 36.9 | 124,785 | 1% |
| *R. saxatilis* | 75.9 | 125,698 | 0% |
| *R. sengorensis* | 75.7 | 125,724 | 0% |
| *R. setosus* | 74.9 | 125,560 | 0% |
| *R. spectabilis* | 75.2 | 125,426 | 0% |
| *R. strigosus* | 74.9 | 124,818 | 1% |
| *R. tephrodes* | 8.9 | 125,337 | 0% |
| *R. thomsonii* | 14.7 | 125,710 | 0% |
| *R. treutleri* | 7.2 | 124,235 | 1% |
| *R. tricolor* | 24.3 | 125,699 | 0% |
| *R. trifidus* | 48.3 | 124,841 | 1% |
| *R. trilobus* | 52 | 125,283 | 0% |
| *R. trivialis* | 11 | 125,687 | 0% |
| *R. ulmifolius* | 38 | 125,701 | 0% |
| *R. ursinus* x  *R. armeniacus 1* | 34.5 | 125,664 | 0% |
| *R. ursinus2* | 1.3 | 106,708 | 15% |
| *R. ursinus3* | 74.8 | 125,597 | 0% |
| *R. ursinus4* | 11.7 | 125,035 | 1% |
| *R. ursinus5* | 8.5 | 125,496 | 0% |
| *R. ursinus* x  *R. armeniacus* *6* | 10.9 | 125,604 | 0% |
| *R. urticifolius* | 38.7 | 125,619 | 0% |
| *W. fragarioides* | 15.8 | 116,194 | 8% |

Supplementary Table S5. Number of loci with paralogs for *Rubus* species having different ploidy.

| Taxon | Ploidy | Number of Loci with Paralogs |
| --- | --- | --- |
| R. ursinus (3) | 12x | 17 |
| R. ursinus (4) | 13x | 8 |
| R. canadensis | 2x | 4 |
| R. coreanus | 2x | 3 |
| R. repens | 2x | 1 |
| R. coriifolius | 2x | 1 |
| R. crataegifolius | 2x | 6 |
| R. cuneifolius | 2x | 4 |
| R. deliciosus | 2x | 3 |
| R. nivalis | 2x | 0 |
| R. eriocarpus | 2x | 5 |
| R. hawaiensis | 2x | 1 |
| R. hispidus | 2x | 0 |
| R. idaeus | 2x | 2 |
| R. illecebrosus | 2x | 3 |
| R. innominatus | 2x | 2 |
| R. alexeterius | 2x | 1 |
| R. lasiococcus | 2x | 1 |
| R. lasiostylus | 2x | 7 |
| R. leucodermis | 2x | 3 |
| R. ellipticus | 2x | 5 |
| R. allegheniensis | 2x | 0 |
| R. palmatus | 2x | 0 |
| R. parviflorus | 2x | 2 |
| R. parvifolius | 2x | 2 |
| R. pedatus | 2x | 4 |
| R. phoenicolasius | 2x | 4 |
| R. pubescens | 2x | 0 |
| R. pungens | 2x | 0 |
| R. arcticus | 2x | 6 |
| R. robustus | 2x | 1 |
| R. rosifolius | 2x | 4 |
| R. niveus | 2x | 0 |
| R. occidentalis | 2x | 1 |
| R. odoratus | 2x | 2 |
| R. setosus | 2x | 7 |
| R. spectabilis | 2x | 1 |
| R. argutus | 2x | 2 |
| R. strigosus | 2x | 5 |
| R. trifidus | 2x | 0 |
| R. trilobus | 2x | 4 |
| R. trivialis | 2x | 0 |
| R. ulmifolius | 2x | 4 |
| R. urticifolius | 2x | 0 |
| W. fragarioides | 2x | 1 |
| R. flagellaris | 4-9x | 16 |
| R. australis | 4x | 13 |
| R. bifrons | 4x | 2 |
| R. caesius | 4x | 18 |
| R. calophyllus | 4x | 2 |
| R. fockeanus | 4x | 11 |
| R. glaucus | 4x | 4 |
| R. gunnianus | 4x | 13 |
| R. humulifolius | 4x | 2 |
| R. ichangensis | 4x | 29 |
| R. laciniatus | 4x | 1 |
| R. lambertianus | 4x | 15 |
| R. lineatus | 4x | 9 |
| R. geoides | 4x | 19 |
| R. sachalinensis | 4x | 2 |
| R. saxatilis | 4x | 9 |
| R. moorei | 4x | 14 |
| R. clinocephalus | 4x | 2 |
| R. nepalensis | 4x | 0 |
| R. pectinarioides | 4x | 9 |
| R. pentagonus | 4x | 2 |
| R. parvus | 4x | 7 |
| R. sengorensis | 4x | 4 |
| R. tephrodes | 4x | 2 |
| R. thomsonii | 4x | 0 |
| R. treutleri | 4x | 3 |
| R. tricolor | 4x | 0 |
| R. armeniacus | 4x | 0 |
| R. assamensis | 4x | 23 |
| R. caucasicus | 4x | 0 |
| R. pensilvanicus | 4x | 1 |
| R. calycinus | 6x | 2 |
| R. glabratus | 6x | 19 |
| R. acanthophyllus | 6x | 14 |
| R. irenaeus | 6x | 20 |
| Logan | 6x | 13 |
| R. loxensis | 6x | 4 |
| R. macraei | 6x | 11 |
| Marion | 6x | 6 |
| R. amphidasys | 6x | 18 |
| R. roseus | 6x | 14 |
| R. ursinus (5) | 6x | 4 |
| R. laegaardii | 6x | 4 |
| Boysen | 7x | 4 |
| R. chamaemorus | 8x | 2 |
| R. ursinus (2) | 8x | 1 |
| R. ursinus x armeniacus (1) | 8x | 15 |
| R. ursinus x armeniacus (6) | 8x | 1 |

Supplementary Table S6. Model statistics for biogeographic analysis implemented in BioGeoBEARS based on ASTRAL-II tree topologies. Table of AIC Akaike Information Criterion (AIC) (Akaike, 1973)

| DEC AIC | DEC+j AIC | DIVALIKE AIC | BAYAREALIKE AIC |
| --- | --- | --- | --- |
| 290.7504 | 278.7822 | 303.4582 | 381.0818 |

Supplementary Table S7. Putative *Rubus* hybrid groups and species. Hybrid origins supported by discordance in nuclear phylogenies and networks. Maternal progenitors inferred from the chloroplast phylogeny.

| **Putative Hybrids** | **Putative parental groups or taxa** |
| --- | --- |
| Group 5 | Putative parents from Group 7 and Groups 3 and 4 (Possibly *Idaeobatus* and *Cylactis*) |
| Group 6 | Subg. *Rubus* $\times$ *Idaeobatus* hybrids |
| *R. macraei* | Subg. *Rubus* $\times$ *Idaeobatus* hybrid |
| *R. glaucus* | Subg. *Rubus* $\times$ *Idaeobatus* hybrid |
| *R. caesius* | Subg. *Rubus* $\times$ *Idaeobatus* hybrid |
| *R. ursinus* | Subg. *Rubus* $\times$ *Idaeobatus* hybrid |
| *R. ursinus* 1 and 6 | *R. ursinus* $\times$ *armeniacus* hybrid |
| *R. humulifolius* | *Idaeobatus* $\times$ *Cylactis* hybrid |
| *R. saxatilis* | Unknown; Maternal parent likely from *Idaeobatus* and may be a black raspberry |
| *R. pentagonus, R. sengorensis, R. thomsonii* | *Idaeobatus* hybrids or closely related to *Malachobatus* and *Dalibardastrum* progenitors |
| 'Logan' | 'Aughinbaugh' (*R. ursinus*) $\times$ 'Red Antwerp' (*R. idaeus*) |
| 'Boysen' | 'Logan' $\times$ 'Austin Mayes' (*R. baileyanus* $\times$ *R. argutus*). *Idaeobatus* is grandparent via 'Logan' |
| 'Marion' | 'Youngberry' $\times$ 'Olallie'. Derived from *R. ursinus* related cultivars. *Rubus idaeus* multiple generations back in pedigree. |

Supplementary Table S8. Number of studied accessions classified in each subgenus. "Other hybrids" include two *R. ursinus* × *armeniacus* hybrids.

| **Subgenus** | **Accessions** |
| --- | --- |
| *Anoplobatus* | 4 |
| *Chamaebatus* | 5 |
| *Chamaemorus* | 1 |
| *Comaropsis* | 1 |
| *Cylactis* | 7 |
| *Dalibarda* | 1 |
| *Dalibardastrum* | 4 |
| *Diemenicus* | 1 |
| *Idaeobatus* | 24 |
| *Malachobatus* | 8 |
| *Micranthobatus* | 3 |
| *Orobatus* | 5 |
| *Rubus* | 24 |
| Horticultural Hybrid | 3 |
| Other Hybrid | 2 |
| Outgroup | 1 |
| **Total** | **94** |
|  |  |
